# Exploring Dysphagia in Congenital Diaphragmatic Hernia: A Retrospective Analysis

**DOI:** 10.3390/pediatric17010003

**Published:** 2025-01-03

**Authors:** Jamie Gilley, Elise Whalen, Audrey Latimore, Viviane Jung, Joseph Hagan, Alice King

**Affiliations:** 1 Department of Pediatrics, Division of Neonatology, Texas Children’s Hospital, Baylor College of Medicine, Houston, TX 77030, USA; 2 Department of Pediatrics, Divisions of Pulmonology and Advanced Practice Providers, Texas Children’s Hospital, Baylor College of Medicine, Houston, TX 77030, USA; 3 Department of Speech, Language, and Learning, Texas Children’s Hospital, Houston, TX 77030, USA; 4 Department of Pediatric Surgery, Division of Pediatric Surgery, Baylor College of Medicine, Houston, TX 77030, USA

**Keywords:** congenital diaphragmatic hernia (CDH), dysphagia, nasogastric tube (NG), gastric tube (GT)

## Abstract

**Background:** Congenital diaphragmatic hernia (CDH) is a complex congenital disorder often accompanied by long-term feeding difficulties. There is a paucity of published data regarding the impact of swallowing difficulties on long-term patient outcomes. Our study attempts to evaluate this phenomenon. **Methods:** A retrospective chart review of infants born with CDH between 2021 and 2022 identified 45 patients. The following variables were identified: need for swallow study, stomach location, defect type, need for anti-reflux therapy, need for nasogastric tube (NG) or gastric tube (GT) at time of discharge, poor growth, and frequency of respiratory infections during the first 12 months of life. **Results:** Thirty-one percent of patients (*n* = 14) underwent a swallow study, 20% (*n* = 9) required long-term anti-reflux medications, 18% (*n* = 8) had a GT and 59% (*n* = 26) had an NG in place at time of discharge, 44% (*n* = 17) experienced poor growth as an outpatient, and 35% (*n* = 16) had respiratory infections in the first 12 months of life requiring hospitalization. Infants with a Type D defect commonly required GT at discharge (40%), experienced respiratory infections in the first 12 months (67%), and had poor growth as an outpatient (67%). **Conclusions:** Our findings underscore the need for routine dysphagia screening in CDH infants during NICU admission. Differences in outcomes based on defect type suggest that early identification and targeted interventions for feeding and swallowing issues may improve long-term growth and respiratory outcomes for CDH patients. Further studies are warranted to develop standardized dysphagia management guidelines for this population.

## 1. Introduction

Infant dysphagia is a complex phenomenon that can impact children beyond the immediate infancy phase and extend into early childhood [1]. Signs of dysphagia can present differently in each patient and may occur in various phases including oral phase (latching problems, reduced expression of milk), pharyngeal phase (coughing during feedings, pharyngeal pooling), and esophageal phase (retrograde flow of milk, and poor motility). Symptoms are often associated with overt or silent tracheal aspiration, gastroesophageal reflux disease (GERD), growth failure, and/or developmental delays. There are several risk factors for dysphagia such as prematurity, GERD, and congenital anomalies, with some infants holding multiple risk factors [1].

CDH is a known risk factor for dysphagia and aspiration [1]. CDH is characterized by the herniation of abdominal contents through the diaphragm into the thoracic cavity while in utero. This herniation results in well described pulmonary hypoplasia, abnormal pulmonary vascular development, and pulmonary hypertension (PH). CDH is estimated to occur in one of every three thousand live births and results in high morbidity and mortality [2]. In severe cases, fetal endoscopic tracheal occlusion or FETO may be offered in an effort to increase fetal lung volume [2]. Despite advances in prenatal diagnosis and imaging, no reliable diagnostic tool currently exists to predict the extent of respiratory complications from CDH such as PH. Postnatal management typically includes respiratory support, blood pressure control, and sedation to optimize oxygenation and ventilation. Even with aggressive postnatal management, infants with severe CDH-PH may require extracorporeal membrane oxygenation [2]. Among those infants who survive to discharge, many continue to face significant challenges including the need for chronic respiratory support, along with feeding difficulties that may contribute to failure to thrive [3].

Children diagnosed with CDH frequently experience GERD, likely due to the positioning of the gastroesophageal junction. Some studies suggest that factors such as intrathoracic liver herniation and antenatal stomach position may further contribute to gastrointestinal issues in these patients [4]. Additionally, animal models of CDH have shown fewer stomach ganglia and reduced peristaltic movement in the esophagus and small intestine, suggesting an inherent motility disorder that could underlie feeding challenges [5]. Dysphagia assessment in infants with CDH is often insufficient, as post-surgical care primarily focuses on symptom-based management, potentially overlooking dysphagia. Diagnosing dysphagia in this population is challenging, as symptoms often resemble those of GERD, which is well documented in CDH patients. Prolonged respiratory support and delayed initiation of oral feeding complicate dysphagia recognition and treatment. Furthermore, some institutions may lack skilled providers, such as occupational therapists (OTs) and speech–language pathologists (SLPs), who are trained to evaluate and diagnose dysphagia [6].

Due to the gap in knowledge of dysphagia in children with CDH, our study aimed to evaluate the prevalence of dysphagia in our cohort and its role in feeding difficulties. We also sought to identify opportunities for earlier feeding interventions to reduce long-term health risks during the child’s initial years of life. Targeted interventions addressing dysphagia in this population are crucial in minimizing further injury to the hypoplastic lung parenchyma and supporting improved growth and development [6].

## 2. Materials and Methods

With Institutional Review Board approval, we conducted a retrospective chart review of infants diagnosed with CDH between 2021 and 2022 who received care at a tertiary children’s hospital through their first 12 months of life. Given the limited research on dysphagia in this population, this study was proposed as a two-year pilot study and intended to provide preliminary data to support a larger, more adequately powered study. This small-scale study was intended to investigate the possible occurrence of dysphagia in the CDH population given the lack of available studies in an effort to bring global awareness to this potential issue and provide preliminary data to guide future studies. All infants born or admitted with a diagnosis of CDH were included in the review. Our institution admits approximately 25–35 CDH patients annually. Exclusion criteria included death before discharge or one year of life. A total of 45 patients were included in the study, with three being excluded due to death before discharge. Demographic data, surgical data, and feeding outcomes including clinical swallow evaluations and video-fluoroscopic swallow studies (VFSSs) were collected and analyzed from 45 patients diagnosed with CDH. Infants who needed a VFSS were initially evaluated by an OT to assess oral motor strength, suck swallow breath patterns, and safety to feed orally. If concerns arose, the OT would recommend referral to a SLP who can assess for risk of aspiration and refer for testing if needed. VFSS necessity was determined by the SLPs bedside feeding assessment utilizing the functional oral intake scale and evaluating for coughing/choking, physiologic instability such as decreased oxygen saturations and/or bradycardia events, increased work of breathing, stress cues demonstrated by the infant during feeding, and/or congestion with feeds. If concerns arose based on the SLPs assessment of the feeding, a referral was made to obtain a VFSS in order to objectively assess the infant’s swallowing physiology and safety [7].

Data captured included gestational age (GA), birth weight, prenatal MRI markers (liver up percentage, total fetal lung volume, observed-to-expected lung-to-head ratio), stomach location, CDH laterality, and defect type (Table 1). Additional variables related to dysphagia risk included the need for a gastrostomy tube (GT) or nasogastric tube (NG), VFSS results, reflux medication use, outpatient poor growth (defined by ICD-10 failure to thrive codes), and frequency of respiratory infections requiring hospitalizations within the first 12 months of life.

Results included defect type, stomach herniation, use of proton pump inhibitors, NG/GT requirements at discharge, outpatient poor growth, and greater than one respiratory infection in the first year of life. These were analyzed to assess dysphagia risk and its association with CDH.

Fisher’s exact test was used to evaluate associations of defect size (A, B, C, D) with categorical variables. Of note, other associated anomalies included an atrial septal defect (*n* = 6), a patent ductus arteriosus (*n* = 1), Simpson–Golabi–Behlmen Syndrome (*n* = 1), and pyloric stenosis (*n* = 1). CDH defect sizes are graded as a type A (diaphragm surrounded by muscle), type B (>50% diaphragm present), type C (<50% diaphragm present), and type D (no or almost no diaphragm) [8]. Multivariable penalized logistic regression was used to examine the association of stomach location with dysphagia after controlling for defect size. Penalized logistic regression analysis was utilized due to low event rates of the outcomes as it performs better than standard logistic regression analysis when there are a small number of events. A separate logistic regression model was fit for each dysphagia characteristic (VFSS, reflux medications, need for NG/GT, outpatient poor growth, and respiratory infections).

## 3. Results

Our retrospective chart review of 45 patients born with CDH revealed that 31% underwent a VFSS (*n* = 14), 20% required reflux medications (*n* = 9), 18% (*n* = 8) had a GT and 58% (*n* = 26) had an NG in place at time of discharge, 44% experienced poor growth as an outpatient (*n* = 17), and 29% had frequent respiratory infections in the first 12 months of life requiring hospitalization (*n* = 13), with eight missing data due to loss of follow up (Table 2). Of the patients who went home with an NG/GT, 15% (*n* = 7) still required a feeding device at one year of life, and one patient required their NG to be converted to a GT. VFSS results revealed the majority of studies took place in the first three months of life (46%), aspiration and/or penetration was noted in 54% of the studies, and all patients required a feeding intervention including a change in nipple flow (62%), pureed feeds (23%), along with GT placement (15%) due to the severity of dysphagia and concern for airway protection.

**Figure 1:**
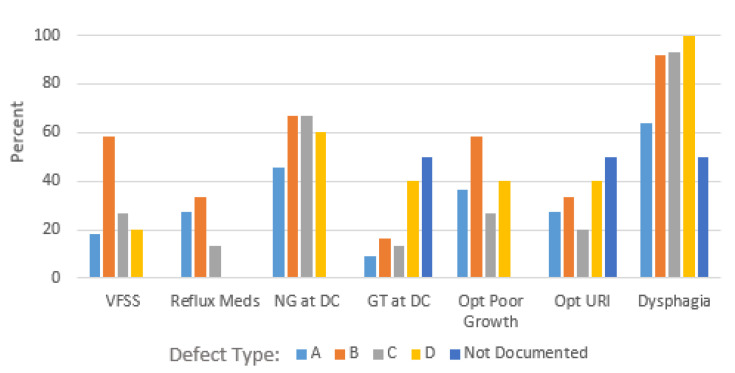
Distribution of outcomes by defect size.

Defect size (A, B, C, D) was not significantly associated with dysphagia (*p* = 0.09), reflux medications (*p* = 0.37), NG (*p* = 0.73) or GT (*p* = 0.53) in place at discharge, outpatient poor growth (*p* = 0.53), or respiratory infections in the first 12 months of life (*p* = 0.75). There were not significant differences in GT at discharge across the four defect type categories (*p* = 0.53), but when we combined all other defect categories, defect type D patients were significantly (*p* = 0.03) more likely to have GT (2/5 = 40%) or NG (3/5 = 60%) at discharge then the other defect types. Defect data were unavailable for two patients, and of the seven right sided type defects, one involved stomach herniation. The distribution of outcomes by defect size are shown in Figure 1.

There were not significant differences in poor growth as an outpatient across the four defect type categories (*p* = 0.54). Note that nine patients were missing data for either poor growth as an outpatient due to loss of follow up and also defect type due to patient demise before repair. There were not significant differences in respiratory infections across the four defect type categories (*p* = 0.59) as noted in Table 3. After controlling for defect size, patients with stomach herniation into the thoracic cavity had significantly higher odds of dysphagia compared to patients with no stomach herniation (adjusted odds ratio = 58.3, 95% confidence interval: 2.6–>1000, *p* = 0.01) as noted in Table 3. No other outcomes were significantly associated with stomach herniation after controlling for defect size. Results were similar when not controlling for defect size, except patients with stomach herniation had significantly higher odds of NG at discharge in unadjusted analysis (odds ratio = 5.1, 95% confidence interval: 1.1–23.6, *p* = 0.04).

## 4. Discussion

Infants born with CDH are at high risk for oropharyngeal dysphagia. Our study’s findings underscore the prevalence of abnormal VFSS results and the need for feeding interventions, such as modified nipple flow or thickened feeds, in this population. Poor growth occurred in almost half of our studied population, with more than a third having frequent respiratory infections in the first 12 months of life. While pulmonary hypoplasia can also contribute to these occurrences, dysphagia could further exacerbate this. Our data suggest infants with a type D defect were more likely to need a GT at discharge, experience poor growth as an outpatient, and have a higher frequency of respiratory infections in the first 12 months of life. Additionally, infants with stomach herniation into the thoracic cavity were more likely to need an NG or GT at time of discharge. Our VFSS results also noted a high risk of aspiration, aligning with findings from Ramaraj et al., where approximately 71% of CDH patients undergoing VFSSs showed aspiration, leading to feeding adjustments in 80% of cases [4].

There are many published studies suggesting the common clinical finding of GERD in this patient population. GERD in the CDH population is a major co-morbidity that affects feeding and nutrition according to Zani et al. [9]. GERD in infants born with CDH is likely related to esophageal dysmotility along with weakness and/or absence of diaphragmatic crura [9]. A systematic review noted a GERD rate of around 53% in infants with CDH and 35% in children with CDH after one year of age [10]. Symptoms of GERD may overlap with oropharyngeal dysphagia, with the evaluation and treatment of these conditions varying greatly in the neonatal population [11]. Additionally, Fishbein et al. noted in their study that oropharyngeal dysphagia is prominent in infants with GERD-like symptoms [12]. Further investigation should be considered to determine the presence of dysphagia in infants with CDH who present with GERD due to their similarity in symptoms that is well described in the literature [13].

There are few published studies addressing swallow dysfunction in infants born with CDH. Two primary studies have investigated the prevalence of aspiration, reporting a high incidence of aspiration on VFSSs within the neonatal CDH population [6]. One study found that the severity of the CDH defect did not correlate with aspiration on VFSSs [10]. In another study, Schwab et al. identified dysphagia in this population and recommended that trained specialists be involved in the care of infants with CDH [6]. Another area to investigate in this population is the concept of esophageal and stomach peristalsis. As noted earlier, CDH animal studies have shown esophageal and small bowel peristalsis, likely linked to fetal development [5]. Arena et al. noted stomach peristalsis in a subgroup of patients who had undergone CDH repair, raising concerns for foregut dysmotility in survivors of CDH [14]. Another study noted infants with CDH who underwent ECMO and had a large defect were prone to severe and retractable reflux, hypothesized to be linked to gastric dysmotility [15]. Further research into stomach and esophageal dysmotility in this population could prove beneficial in determining if CDH defect type and size play a role in foregut dysmotility.

This study has brought to light a potential need in the CDH population. Early interventions by trained specialists, such as occupational therapists and SLPs, could help to identify early feeding and swallowing issues when oral feeds are being initiated. Power et al. describes the various feeding issues survivors of CDH encounter, which may not be adequately supported clinically [16]. Objective swallow assessments may be crucial for developing the safest feeding plan as these assessments provide visual evidence of aspiration, including silent aspiration, which is more common in the pediatric population [11]. Our study’s findings align with the literature regarding the risk of silent aspiration in this population and the need to evaluate swallowing difficulties early utilizing VFSSs. The consequences of silent aspiration can include long-term lung injury, compromised immune response, chronic airway disease, and feeding difficulties [17]. Swallow assessments should be strongly considered by the medical team, particularly if clinical exams reveal signs of swallow dysfunction. Early interventions by feeding specialists when clinically able, along with outpatient follow up, may be crucial to help infants born with CDH to meet the nutritional needs to achieve adequate growth and decrease long term morbidity. There were several limitations to this study. A methodological limitation of the present study is the small sample size, leading to an underpowered study, which informs the need for a larger more adequately powered study. We acknowledge that penalized regression methods are not a powerful tool in small sample sizes and can result in wide confidence intervals, which further demonstrates the need for a larger sample size study. The small sample size precluded adjustment for multiple covariates when comparing outcomes for CDH patients with versus without stomach herniation, however we felt that it was important to control for defect size when making these comparisons despite the fact that the resulting confidence intervals were quite wide. There should be at least 10 events per predictor when performing binary multivariable logistic regression analysis [18], but for the outcomes compared for CDH patients with versus without stomach herniation, only NG at discharge satisfied this criterion. Due to the low event rates of the outcomes compared, we used penalized logistic regression analysis which performs better than standard logistic regression analysis when there are a small number of events [19].

Patient follow-up challenges also presented limitations, as some patients were unable to follow up due to out-of-state travel, resulting in missing data, and one patient was unable to complete an outpatient VFSS. Our study also did not take into account external causes contributing to dysphagia and lung issues in this population, such as infection [20]. Despite these limitations, our study demonstrates a clear clinical need for managing feeding difficulties and assessing dysphagia in CDH patients.

We evaluated five parameters associated with dysphagia (VFSS need, reflux medications, GT placement, outpatient poor growth, and respiratory infections). Poor growth, reflux, and frequent respiratory infections are the most common long-term complications in CDH survivors [13]. Future studies assessing additional dysphagia factors, such as delayed initiation of oral feeds, oral aversion, prolonged intubation, extended hospitalization, and esophageal or gastric dysmotility, could offer further insight into addressing dysphagia in this critical population.

## 5. Conclusions

The results of this study show high risk of aspiration for infants born with CDH who underwent VFSSs. Our results suggest dysphagia could be an additional driver of poor growth in the CDH population with patient-specific, variable symptoms. Additionally, our study demonstrated infants with stomach herniation were more likely to require an NG or GT at time of discharge. Early identification of feeding difficulties in the neonatal period by trained feeding specialists and incorporation of VFSSs to evaluate for aspiration could prove beneficial in this population by modifying feeding strategies to help with overall growth and development. Additional studies with a larger sample size are needed to further evaluate and characterize dysphagia in this population.

## Figures and Tables

**Table 1:** Demographic data.

**Gender:**		**Defect Side:**	
Male	30	Left	37
Female	15	Right	8
**GA:**		**FETO:**	
<35 wks	12	Yes	6
37–39 wks	20	No	39
>39 wks	13		
**Birthweight:**		**Defect Type:**	
1500–2000 gms	1	A	11
2000–2500 gms	3	B	12
2500–3000 gms	13	C	15
3000–3500 gms	22	D	5
>3500 gms	6	Not Documented	2
**Liver Up:**		**Patch:**	
Yes	28	Yes	25
No	17	No	20
**ECMO:**			
Yes	8		
No	37		

**Table 2:** Frequencies and percentages of patients born with CDH that evaluated the need for a swallow study (VFSS), reflux medications, GT placement, poor growth as an outpatient, and outpatient upper respiratory infections (URIs) during the first 12 months of life.

	Frequency (%)
VFSS	14 (31.1)
GT at discharge	8 (17.8)
NG at discharge	26 (57.8)
Outpatient URI	
Yes	13 (28.9)
No	24 (53.3)
Unknown	8 (17.8)
Reflux medications	9 (20.0)
Outpatient poor growth	
Yes	20 (44.4)
No	17 (37.8)
Unknown	8 (17.8)

**Table 3:** Comparison of outcomes for CDH patients with no stomach herniation versus stomach herniation.

Outcome	Odds Ratio	95% Confidence Interval		*p*-Value	Adjusted Odds Ratio ^1^	Adjusted 95% Confidence Interval ^1^		Adjusted *p*-Value ^1^
VFSS	15.334	0.719	327.190	0.08	10.343	0.555	192.806	0.12
Reflux Meds	2.291	0.318	16.495	0.41	7.808	0.237	257.409	0.35
NG at DC	5.074	1.090	23.613	0.04	4.384	0.884	21.736	0.07
GT at DC	6.426	0.293	141.041	0.24	6.135	0.329	114.395	0.22
Opt Poor Growth	4.694	0.869	25.349	0.07	3.585	0.624	20.602	0.15
Opt URI	4.586	0.633	33.208	0.13	4.483	0.574	35.042	0.15
Dysphagia	63.001	2.846	>999.999	0.01	58.298	2.550	>999.999	0.01

^1^ Adjusted for defect size.

## Data Availability

All data generated or analyzed during this study are included in this article. Further inquiries can be directed to the corresponding author.
